# Guidance concerning chiropractic practice in response to COVID-19 in the U.S.: a summary of state regulators’ web-based information

**DOI:** 10.1186/s12998-020-00333-6

**Published:** 2020-07-06

**Authors:** Shawn M. Neff, Christopher B. Roecker, Casey S. Okamoto, Samuel L. Holguin, Jason G. Napuli, Ross Mattox, Nathan A. Hinkeldey, David J. Paris

**Affiliations:** 1 Martinsburg Veterans Affairs Medical Center, Martinsburg, WV USA; 2 Veterans Affairs Nebraska-Western Iowa Health Care System, Grand Island, NE USA; 3 Minneapolis Veterans Affairs Medical Center, Minneapolis, MN USA; 4 St Louis Veterans Affairs Health Care System, St Louis, MO USA; 5 Veterans Affairs Central Iowa Health Care System, Des Moines, IA USA; 6 Veterans Affairs Northern California Health Care System, Redding, CA USA

**Keywords:** Chiropractic, COVID-19, Coronavirus disease 2019, 2019 novel coronavirus disease, Licensure, Governing board, Regulation, Social control

## Abstract

**Introduction:**

The COVID-19 pandemic led to unprecedented changes, as many state and local governments enacted stay-at-home orders and non-essential businesses were closed. State chiropractic licensing boards play an important role in protecting the public via regulation of licensure and provision of guidance regarding standards of practice, especially during times of change or uncertainty.

**Objective:**

The purpose of this study was to summarize the guidance provided in each of the 50 United States, related to chiropractic practice during the COVID-19 pandemic.

**Methods:**

A review of the public facing websites of governors and state chiropractic licensing boards was conducted in the United States. Data were collected regarding the official guidance provided by each state’s chiropractic licensing board as well as the issuance of stay-at-home orders and designations of essential personnel by state governors. Descriptive statistics were used to report the findings from this project.

**Results:**

Each of the 50 state governor’s websites and individual state chiropractic licensing board’s websites were surveyed. Stay-at-home or shelter-in-place orders were issued in 86% of all states. Chiropractors were classified as essential providers in 54% of states, non-essential in one state (2%), and no guidance was provided in the remaining 44% of all states. Fourteen states (28%) recommended restricting visits to only urgent cases and the remaining states (72%) provided no guidance. Twenty-seven states (54%) provided information regarding protecting against infectious disease and the remaining states (46%) provided no guidance. Twenty-two states (44%) provided recommendations regarding chiropractic telehealth and the remaining states (56%) provided no guidance. Seventeen states (34%) altered license renewal requirements and eight states (16%) issued warnings against advertising misleading or false information regarding spinal manipulation and protection from COVID-19.

**Conclusion:**

State guidance during the COVID-19 pandemic was heterogenous, widely variability in accessibility, and often no guidance was provided by state chiropractic licensing boards. Some state chiropractic licensing boards chose to assemble guidance for licensees into a single location, which we identified as a best practice for future situations where changes in chiropractic practice must be quickly communicated.

## Introduction

In December 2019, a novel coronavirus (SARS-CoV-2) was identified as it spread within China and described as causing coronavirus disease 2019 (COVID-19). This infectious disease spread quickly around the globe; the World Health Organization declared COVID-19 to be a Public Health Emergency of International Concern in January, 2020 and a pandemic in March of 2020 [1]. The first case of COVID-19 was reported in the United States (U.S.) on January 21, 2020 and by April 10, 2020 there were approximately 500,000 confirmed cases in the U.S. and over 1.5 million cases, worldwide [2, 3]. These statistics should be considered along with widespread scarcity of testing supplies and frequent testing delays [4], which likely resulted in underestimation of the true prevalence of COVID-19 [5].

In an effort to slow the spread of COVID-19 and reduce strain on the U.S. healthcare system, various U.S. state governments offered guidance in the form of stay-at-home orders. These orders outlined how travel should be limited to essential purposes, such as obtaining food or reporting for essential employment, but often amounted to recommendations, rather than enforceable mandates [6]. State governors were instructed to make independent decisions regarding their respective state’s response to the COVID-19 pandemic [7]. This led to vastly different responses among the various states [8]. In the U.S. chiropractic, like most health professions, is regulated at the state level, not at the national level [9]. Chiropractors were described as essential healthcare workers in a memo by the U.S. Department of Homeland Security made available on March 28, 2020 [10]. Since the nature of this memo was advisory, rather than a formal federal directive, chiropractors were left to rely on their state’s board of chiropractic examiners (i.e. state licensing board or state board) for direction [11]. At a time when our healthcare system is stressed to its limits, doctors of chiropractic have been described as serving to mitigate the demand of musculoskeletal pain patients on primary care providers, urgent care providers, and emergency departments [12]. The benefit of providing this service must be balanced with the public health risks that come with providing direct patient care and potentially increasing the spread of COVID-19 [13].

The objective of this study was to summarize the guidance provided in each of the 50 states related to chiropractic practice during the COVID-19 pandemic and to report these results using descriptive statistics.

## Methods

Websites for each state governor’s office as well as each state’s chiropractic licensing board were searched between April 3, 2020 and April 10, 2020. These websites were manually searched for guidance related to the status of chiropractic practice during the COVID-19 pandemic. Any changes made to these public websites after April 10, 2020 were not captured and, therefore, not included within this report. Changes in practice regulations based on COVID-19 that were made before 4/3/2020 and deleted or amended between that date and the study close date were also not included in this study. Information obtained and reported within this report was limited to statements directly from, or hyperlinked to, the state governor’s or state chiropractic licensing board’s websites. Governors’ websites were accessed using internet search engines (e.g. Google searches) while state chiropractic boards’ websites were accessed via hyperlinks provided by the Federation of Chiropractic Licensing Board’s online directory.

Seven policy domains relevant to chiropractic practice during the COVID-19 pandemic were identified via consensus by the authors of this report. These 7 domains were established by attempting to anticipate the most relevant guidance necessary to inform general chiropractic practice for doctors of chiropractic located throughout the United States. The seven domains involve: 1.) shelter-in-place or stay-at-home orders/directives, 2.) classification of chiropractors as essential healthcare providers, 3.) restriction of chiropractic practice to urgent/emergent presentations, 4.) recommendations for infectious disease control or use of personal protective equipment (PPE), 5.) chiropractic telehealth recommendations, 6.) alterations to continuing education (CE) or license renewal requirements (e.g. deadline extensions or changes to distance learning limitations), and 7.) warnings against false, deceptive, or misleading claims related to spinal manipulation/adjustments conferring protection against infection or COVID-19.

In an attempt to capture all relevant policy information and recommendations relevant to this project, a minimum of two authors independently reviewed each of the seven domains involved with this project for each of the 50 United States. Any disagreements or ambiguities were discussed with the remaining authors and determinations were made based on consensus discussion and majority vote. These ambiguities were not common and usually involved information that was not directly provided by state board’s websites, but was able to be discovered after a meandering path of multiple hyperlinks were followed to identify the relevant information. For pragmatic reasons, this project was limited to only including information that was available within two or fewer hyperlinks from the original governor’s website or state chiropractic licensing board’s website.

## Results

All 50 state chiropractic licensing boards and governor’s websites were accessed and reviewed as part of this project (see Appendix A, Additional File 1). Results from each of this survey’s 7 domains were reported for each state in Tables 1, 2, 3 and 4.

**Table 1:** Stay-at-Home Orders and Classification as “Essential” Healthcare Provider Status for Each of the 50 United States During the COVID-19 Pandemic

State	Stay-at-Home or Shelter-in-Place Order	Chiropractors Classified as Essential
Alabama	Yes, enacted on 04/04/2020	Yes, per State Board
Alaska	Yes, enacted on 03/28/2020	No Guidance from State Board
Arizona	Yes, enacted on 03/31/2020	Yes, per State Board Governor
Arkansas	None Issued	Yes, per State Board and Governor
California	Yes, enacted on 03/19/2020	No Guidance from State Board
Colorado	Yes, enacted on 03/26/2020	No Guidance from State Board
Connecticut	Yes, enacted on 03/23/2020	No Guidance from State Board
Delaware	Yes, enacted on 03/24/2020	No Guidance from State Board
Florida	Yes, enacted on 04/03/2020	Yes, per Governor
Georgia	Yes, enacted on 04/03/2020	Yes, per State Board
Hawaii	Yes, enacted on 03/25/2020	No Guidance from State Board
Idaho	Yes, enacted on 03/25/2020	Yes, per Governor
Illinois	Yes, enacted on 03/21/2020	Yes, per Governor
Indiana	Yes, enacted on 03/24/2020	Yes, per Governor
Iowa	None Issued	No Guidance from State Board
Kansas	Yes, enacted on 03/30/2020	Yes, per Governor
Kentucky	Yes, enacted on 03/26/2020	No, determined to be non-essential
Louisiana	Yes, enacted on 03/23/2020	No Guidance from State Board
Maine	Yes, enacted on 04/02/2020	Yes, per Governor
Maryland	Yes, enacted on 03/30/2020	Yes, per State Board (via CISA guidance)
Massachusetts	Yes, enacted on 03/24/2020	Yes, per BCE
Michigan	Yes, enacted on 03/24/2020	Yes, per Governor
Minnesota	Yes, enacted on 03/27/2020	Yes, per State Board
Mississippi	Yes, enacted on 04/03/2020	Yes, per Governor
Missouri	Yes, enacted on 04/06/2020	Yes, per Governor
Montana	Yes, enacted on 03/28/2020	No Guidance from State Board
Nebraska	None Issued	No Guidance from State Board
Nevada	Yes, enacted on 04/01/2020	No Guidance from State Board
New Hampshire	Yes, enacted on 03/27/2020	No Guidance from State Board
New Jersey	Yes, enacted on 03/21/2020	No Guidance from State Board
New Mexico	Yes, enacted on 03/24/2020	Yes, per Governor
New York	Yes, enacted on 03/22/2020	No Guidance from State Board
North Carolina	Yes, enacted on 03/30/2020	No Guidance from State Board
North Dakota	None Issued	No Guidance from State Board
Ohio	Yes, enacted on 03/23/2020	Yes, per State Board
Oklahoma	None Issued	Yes, per State Board
Oregon	Yes, enacted on 03/23/2020	Yes, per Oregon Health Authority
Pennsylvania	Yes, enacted on 04/01/2020	Yes, per State Board and Governor
Rhode Island	Yes, enacted on 03/28/2020	No Guidance from State Board
South Carolina	Yes, enacted on 04/07/2020	Yes, per Governor
South Dakota	None Issued	No Guidance from State Board
Tennessee	Yes, enacted on 03/31/2020	Yes, per Governor
Texas	Yes, enacted on 04/02/2020	Yes, per State Board
Utah	Yes, enacted on 03/27/2020	No Guidance from State Board
Vermont	Yes, enacted on 03/25/2020	No Guidance from State Board
Virginia	Yes, enacted on 03/30/2020	No Guidance from State Board
Washington	Yes, enacted on 03/23/2020	Yes, per State Board
West Virginia	Yes, enacted on 03/24/2020	Yes, per State Board
Wisconsin	Yes, enacted on 03/25/2020	Yes, per Wisconsin’s Department of Safety and Professional Services (DSPS)
Wyoming	None Issued	No Guidance from State Board

**Table 2:** Practice Restrictions, Hosting Patient Safety Information, and Telehealth Recommendations for Each of the 50 United States During the COVID-19 Pandemic

State	Restrictions on Chiropractic Patients to Emergency/Urgent Only	Information Provided Regarding Protective Equipment and/or Patient Safety	Recommendations Regarding Chiropractors Providing Telehealth Services
Alabama	Restricted Practice	Yes, Information was Provided	Yes, Not Eligible for Telehealth
Alaska	No practice restrictions	No Guidance from State Board	No Guidance from State Board
Arizona	No practice restrictions	Yes, Information was Provided	Yes, Eligible for Telehealth
Arkansas	No practice restrictions	Yes, Information was Provided	No Guidance from State Board
California	Restricted Practice	Yes, Information was Provided	No Guidance from State Board
Colorado	No practice restrictions	No Guidance from State Board	No Guidance from State Board
Connecticut	No practice restrictions	Yes, Information was Provided	No Guidance from State Board
Delaware	No practice restrictions	No Guidance from State Board	No Guidance from State Board
Florida	No practice restrictions	Yes, Information was Provided	Yes, Eligible for Telehealth
Georgia	No practice restrictions	Yes, Information was Provided	Yes, Eligible for Telehealth
Hawaii	No practice restrictions	No Guidance from State Board	No Guidance from State Board
Idaho	No practice restrictions	No Guidance from State Board	No Guidance from State Board
Illinois	Restricted Practice	Yes, Information was Provided	Yes, Eligible for Telehealth
Indiana	No practice restrictions	No Guidance from State Board	No Guidance from State Board
Iowa	No practice restrictions	No Guidance from State Board	No Guidance from State Board
Kansas	No practice restrictions	Yes, Information was Provided	Yes, Eligible for Telehealth
Kentucky	No practice restrictions	No Guidance from State Board	No Guidance from State Board
Louisiana	No practice restrictions	No Guidance from State Board	Yes, Eligible for Telehealth
Maine	Restricted Practice	No Guidance from State Board	Yes, Eligible for Telehealth
Maryland	Restricted Practice	Yes, Information was Provided	No Guidance from State Board
Massachusetts	No practice restrictions	Yes, Information was Provided	Yes, Eligible for Telehealth
Michigan	No practice restrictions	Yes, Information was Provided	Yes, Eligible for Telehealth
Minnesota	Restricted Practice	Yes, Information was Provided	Yes, Eligible for Telehealth
Mississippi	No practice restrictions	No Guidance from State Board	No Guidance from State Board
Missouri	No practice restrictions	No Guidance from State Board	No Guidance from State Board
Montana	No practice restrictions	No Guidance from State Board	No Guidance from State Board
Nebraska	No practice restrictions	No Guidance from State Board	No Guidance from State Board
Nevada	No practice restrictions	Yes, Information was Provided	No Guidance from State Board
New Hampshire	No practice restrictions	No Guidance from State Board	No Guidance from State Board
New Jersey	No practice restrictions	No Guidance from State Board	No Guidance from State Board
New Mexico	No practice restrictions	No Guidance from State Board	No Guidance from State Board
New York	No practice restrictions	Yes, Information was Provided	Yes, Eligible for Telehealth
North Carolina	No practice restrictions	No Guidance from State Board	Yes, Eligible for Telehealth
North Dakota	No practice restrictions	No Guidance from State Board	No Guidance from State Board
Ohio	No practice restrictions	Yes, Information was Provided	No Guidance from State Board
Oklahoma	Restricted Practice	Yes, Information was Provided	No Guidance from State Board
Oregon	Restricted Practice	Yes, Information was Provided	Yes, Eligible for Telehealth
Pennsylvania	Restricted Practice	Yes, Information was Provided	Yes, Eligible for Telehealth
Rhode Island	No practice restrictions	No Guidance from State Board	No Guidance from State Board
South Carolina	No practice restrictions	Yes, Information was Provided	Yes, Eligible for Telehealth
South Dakota	No practice restrictions	Yes, Information was Provided	No Guidance from State Board
Tennessee	Restricted Practice	Yes, Information was Provided	No Guidance from State Board
Texas	Restricted Practice	Yes, Information was Provided	Yes, Eligible for Telehealth
Utah	No practice restrictions	Yes, Information was Provided	Yes, Eligible for Telehealth
Vermont	Restricted Practice	No Guidance from State Board	Yes, Eligible for Telehealth
Virginia	No practice restrictions	No Guidance from State Board	Yes, Eligible for Telehealth
Washington	Restricted Practice	Yes, Information was Provided	Yes, Eligible for Telehealth
West Virginia	No practice restrictions	Yes, Information was Provided	No Guidance from State Board
Wisconsin	No practice restrictions	Yes, Information was Provided	Yes, Eligible for Telehealth
Wyoming	No practice restrictions	No Guidance from State Board	No Guidance from State Board

**Table 3:** Alterations to Chiropractic State Licensure or License Recertification for Each of the 50 United States During the COVID-19 Pandemic

State	Alterations to Chiropractic State Licensure or Recertification Requirements
Alabama	No Changes Reported
Alaska	No Changes Reported
Arizona	No Changes Reported
Arkansas	No Changes Reported
California	Waived Requirements
Colorado	No Changes Reported
Connecticut	Waived Requirements
Delaware	Extended Deadlines
Florida	Extended Deadlines
Georgia	No Changes Reported
Hawaii	No Changes Reported
Idaho	No Changes Reported
Illinois	Accepted All Online Continuing Education Credits
Indiana	Extended Deadlines
Iowa	Waived In-Person Requirements
Kansas	No Changes Reported
Kentucky	No Changes Reported
Louisiana	No Changes Reported
Maine	Waived Requirements or Deadline Extension
Maryland	No Changes Reported
Massachusetts	Extended Deadlines
Michigan	Extended Deadlines
Minnesota	No Changes Reported
Mississippi	No Changes Reported
Missouri	No Changes Reported
Montana	No Changes Reported
Nebraska	No Changes Reported
Nevada	No Changes Reported
New Hampshire	No Changes Reported
New Jersey	No Changes Reported
New Mexico	Extended Deadlines
New York	Accepted All Online Continuing Education Credits
North Carolina	No Changes Reported
North Dakota	No Changes Reported
Ohio	No Changes Reported
Oklahoma	No Changes Reported
Oregon	No Changes Reported
Pennsylvania	No Changes Reported
Rhode Island	No Changes Reported
South Carolina	Extended Deadlines
South Dakota	No Changes Reported
Tennessee	No Changes Reported
Texas	No Changes Reported
Utah	Accepted All Online Continuing Education Credits
Vermont	Accepted All Online Continuing Education Credits and Extended Deadlines
Virginia	No Changes Reported
Washington	Accepted All Online Continuing Education Credits
West Virginia	Accepted All Online Continuing Education Credits
Wisconsin	No Changes Reported
Wyoming	Accepted All Online Continuing Education Credits

**Table 4:** State Chiropractic Boards’ Guidance Regarding Misinformation During the COVID-19 Pandemic

State	Misinformation Guidance
Alabama	No guidance from State Board
Alaska	No guidance from State Board
Arizona	Yes, provided warning against misinformation
Arkansas	Yes, provided warning against misinformation
California	No guidance from State Board
Colorado	No guidance from State Board
Connecticut	No guidance from State Board
Delaware	No guidance from State Board
Florida	No guidance from State Board
Georgia	No guidance from State Board
Hawaii	No guidance from State Board
Idaho	No guidance from State Board
Illinois	No guidance from State Board
Indiana	No guidance from State Board
Iowa	No guidance from State Board
Kansas	No guidance from State Board
Kentucky	No guidance from State Board
Louisiana	No guidance from State Board
Maine	No guidance from State Board
Maryland	No guidance from State Board
Massachusetts	No guidance from State Board
Michigan	No guidance from State Board
Minnesota	Yes, provided warning against misinformation
Mississippi	No guidance from State Board
Missouri	No guidance from State Board
Montana	No guidance from State Board
Nebraska	No guidance from State Board
Nevada	No guidance from State Board
New Hampshire	No guidance from State Board
New Jersey	No guidance from State Board
New Mexico	No guidance from State Board
New York	No guidance from State Board
North Carolina	No guidance from State Board
North Dakota	No guidance from State Board
Ohio	Yes, provided warning against misinformation
Oklahoma	No guidance from State Board
Oregon	Yes, provided warning against misinformation
Pennsylvania	No guidance from State Board
Rhode Island	No guidance from State Board
South Carolina	Yes, provided warning against misinformation
South Dakota	No guidance from State Board
Tennessee	No guidance from State Board
Texas	Yes, provided warning against misinformation
Utah	No guidance from State Board
Vermont	No guidance from State Board
Virginia	No guidance from State Board
Washington	No guidance from State Board
West Virginia	Yes, provided warning against misinformation
Wisconsin	No guidance from State Board
Wyoming	No guidance from State Board

Forty-three states (86%) issued shelter-in-place (SIP) or stay-at-home (SAH) orders in response to the COVID-19 pandemic, while 7 states (14%) did not (see Table 1). Two states (Arkansas and Oklahoma) did not issue SIP/SAH orders, but did describe chiropractors as essential healthcare providers. The remaining states without SIP/SAH orders offered little guidance on any of the 7 domains. In the absence of SIP/SAH orders, guidance regarding chiropractic practice may have been considered unnecessary.

Guidance regarding chiropractors’ status as essential healthcare providers was provided by 27 states (54%); 26 of these states classified chiropractors as essential, while one state (Kentucky) expressly stated that chiropractors were non-essential [14].

The remaining 23 states (46%) failed to provide guidance regarding whether or not chiropractors were considered essential in their respective states (see Table 1). In this study, we classified chiropractors in Colorado as essential. On March 19, 2020 an executive order by the Colorado governor was issued, ordering all chiropractic clinics to close, unless they were operating within a medical facility and restricting visits to only urgent/emergency situations. On April 6, 2020 the Colorado governor reversed that order and permitted community-based chiropractors to resume clinical practice in situations where delaying care may result in rapid progression of the patient’s condition or deterioration of the patient’s health [15, 16].

Guidance varied regarding whether chiropractors were to maintain “business as usual” or restrict their face-to-face clinical practice to only those patient interactions which constituted urgent, acute, or emergency patient care (i.e. restricted practice). Fourteen state chiropractic licensing boards (28%) provided guidance to restrict face-to-face chiropractic appointments to only those patients deemed to have urgent, acute, or emergency conditions; the remaining 36 states (72%) provided no guidance on whether chiropractors should continue with business as usual or restrict their practices (see Table 2).

Guidance regarding physical distancing and the use of personal protective equipment (PPE) has been provided at a national level by the Centers for Disease Control and Prevention (CDC) [5], and such information may be customized and disseminated by chiropractic state boards to meet specific state and professional requirements. Twenty-seven state chiropractic boards (54%) provided information, or hosted links to information, regarding patient safety or PPE; the remaining 23 state boards (46%) provided no guidance regarding patient safety or the use of PPE (see Table 2).

Telehealth is the delivery of healthcare services via the use of telecommunication technologies and allows for remote patient care, including screening for red flags, providing patient education, and recommending self-care activities. Twenty-two state chiropractic licensing boards (44%) provided guidance on whether chiropractors were appropriate for providing telehealth services, in their respective states. Of the 22 states that provided telehealth guidance, 21 states indicated that chiropractors were eligible to provide telehealth services, while one state (Alabama) indicated that that chiropractors were ineligible to provide telehealth services [17]. The remaining 28 state chiropractic boards (56%) did not provide guidance regarding chiropractors’ ability to serve the individuals in their state, via telehealth (see Table 2).

Alterations in continuing education (CE) requirements or license renewal requirements may be appropriate during the COVID-19 pandemic, due to disrupted travel and widespread cancelations of academic conferences. A total of 17 state chiropractic licensing boards (34%) provided information regarding such CE or license renewal alterations (see Table 3). Alterations included the following: 7 states increased the allowed number of online credit hours to allow for all of the annual CE credits to be earned from online sources, 8 states extended their CE deadlines, 1 state (Vermont) accepted all CE credits from online sources while also extending CE deadlines, and 2 states (California and Connecticut) waived their annual CE requirements.

State chiropractic licensing boards are responsible for protecting the health, welfare, and safety of the public through licensure, education, and enforcement. That responsibility includes protecting patients from public health misinformation. In response to unsubstantiated claims and advertisements from chiropractors regarding the clinical effects of spinal manipulation/adjustments on immune function, some state chiropractic boards issues warning against providing unsubstantiated information. A total of 8 state chiropractic licensing boards (16%) issued warnings against making false, deceptive, or misleading statements about spinal manipulation and its influence on immune function or inferring that spinal manipulation provides protection from COVID-19 (see Table 4).

## Discussion

Pandemics, while infrequent, necessitate timely communication in order to ensure that the public, along with licensed healthcare providers, have the information needed to keep themselves, as well as others safe. Uncertainty regarding the various aspects of COVID-19 has made it difficult for leaders to forecast the overall effect and generate effective safety recommendations. Some of the state chiropractic boards utilized a format that dramatically improved our ability to discover COVID-19-related information for their respective states. These involved calling the user’s attention, often using a banner or other indicator, to a dedicated website or area of their main website containing consolidated information related to the ongoing COVID-19 pandemic. The dedicated websites provided a single location for licensees, or members of the public, to quickly and easily acquire necessary information. Often the dedicated site provided a summary of the most relevant information while providing hyperlinks to more detailed source information (e.g. governor’s stay-at-home order). Visitors of these websites were encouraged to return regularly to this site for updates and included the date and time the website was last updated; occasionally, newly added information was highlighted to aid in identifying recent changes. These sites were perceived as having the user of the website in mind and were designed to be easily discovered, easily interpreted, and to have maximum overall utility. This model was identified by the authors of this manuscript as a “best practice” when attempting to inform state licensees and the general public about guidance or recommendations. For the majority of states, the authors of this project were left having to search disparate websites to locate fragmented information related to chiropractic practice during the COVID-19 pandemic. Having a single site, with consolidated information, seems to reduce the time required to access key information and ensure that all relevant information is communicated from the licensing body to the licensee.

The COVID-19 pandemic emerged in the midst of a global pain crisis and opioid epidemic, complicating clinical decision making [18]. Chiropractors and other providers must balance a responsibility to limit contagion with their responsibility to provide access to pain management, which some organizations have deemed a fundamental right [19, 20]. The clear need for ongoing pain management, in some form, likely contributed to the nearly universal designation of chiropractors as essential healthcare providers. As portal-of-entry providers, chiropractors can perform triage, evaluation, management, differential diagnosis, deliver treatment, or coordinate necessary referral. There may be advantages to seeking care for musculoskeletal complaints at a chiropractic office as opposed to an emergency department. Such advantages include reduced risk of COVID-19 transmission from those presenting to the ED with upper respiratory symptoms as well as conservation of ED resources.

In the 28% of states where chiropractic was deemed an essential healthcare service, the state chiropractic licensing boards uniformly provided guidance that chiropractic care was to be restricted to urgent, acute, or emergent presentations. The definition of “urgent or emergent” is not entirely clear [21]. Differences in patient [22] and professional [23, 24] perception of what constitutes an urgent or emergent situation could result in variable interpretation and practice behaviors. There are several avenues by which the urgency of a condition may be assessed. Low back pain is one of leading complaints evaluated in U.S. emergency departments, accounting for 4.4% of all visits [25]. A review of more than 40,000 patient visits revealed that 2.5–5.1% of patients required immediate attention for spinal pain complaints. The presence of red flags increases the likelihood that patients may have more urgent or serious conditions, such as fracture, cancer, infection, or vascular complication [26]. One can elicit a history and answers to red flag screening questions via telephone, allowing for triage if red flags are present or reassurance if red flags are absent. Atlas and Deyo cited several reasons to consider in-person evaluation, including the presence of any red flags, the presence of radicular symptoms, persistence of symptoms beyond 2 weeks, or if a patient desires in-person evaluation despite reassurance [27]. Telehealth and triage are not methods traditionally used by chiropractors; however, chiropractors are certainly capable of adapting to provide such services, if permitted. Twenty-two state chiropractic licensing boards supported chiropractic implementing telehealth services, 1 state board recommended against the use of such services, and 27 state boards failed to provide guidance. Without clear guidance from their state board, chiropractors are left with uncertainty regarding appropriate clinical practice in the setting of a public health crisis. Such uncertainty may delay care to the public or place the provider at risk of disciplinary action if utilizing a service that is not recommended.

The COVID-19 pandemic represents a global health crisis where there are currently no effective vaccines, treatments, or cures [28]. Therefore, public health measures aimed to minimize the transmission of this viral pathogen, such as practicing good hand hygiene, maintaining physical distancing, and wearing face masks lie at the heart of limiting the spread of this condition. Reports of reduced susceptibility to or recovery from infectious disease following spinal manipulation surfaced during the 1918 influenza pandemic [29–31]. Interest in such a connection has persisted in segments of the chiropractic and osteopathic professions over the past 100 years [32, 33]. Research investigating the relationship between spinal manipulation and immune function has been limited to basic science (non-clinical) studies, involving small sample sizes, and deemed insufficient to validate such claims [34–38]. As the world struggles with a novel virus that has no known treatment or cure, notions of immunomodulation through spinal manipulation have resurfaced on social media, which led to national and international chiropractic organizations providing guidance to their membership. Currently, the American Chiropractic Association (ACA), the International Chiropractic Association (ICA), and the World Federation of Chiropractic (WFC) have all issued statements indicating there is no evidence that spinal manipulation/adjustments have been shown to influence the prevention or treatment of COVID-19 [33, 38, 39]. Additionally, “a united statement of the global chiropractic research community against the pseudoscientific claim that chiropractic care boosts immunity” was signed by approximately 150 researchers [28]. At the time of our data collection only 16% of state boards of chiropractic had made statements regarding false, deceptive, or misleading statements. It is unclear why the majority of state boards had not chosen to issue statements, but that may change as the pandemic progresses and such claims continue to attract unfavorable media attention. Infection rates have differed drastically from state-to-state which may have impacted decision making and resulted in variability regarding the content and timing of board guidance.

## Limitations

There are many limitations associated with this study. It is possible that Governors or state chiropractic licensing boards posted information that was missed during our search. Every attempt was made to thoroughly review each website and capture relevant information, but due to variations in the ways content may have been described or variations in how relevant material may have been hyperlinked, it is possible that information was overlooked and omitted from this report. State chiropractic licensing boards may have also communicated information to their constituents via methods other than their website (e.g., mail, e-mail, or social media). For pragmatic reasons, the methods of this project were limited to reviewing public facing websites. Lastly, information made available after April 10, 2020 was not captured or reported as part of this project. It is possible that updates were in development at the time our search was performed, but had not yet been made publicly available or were only available via direct personal communication with personnel associated with each state’s chiropractic licensing board. Unfortunately, personally communicating with every state board was unfeasible for this project and information obtainable only through direct personal communication was not included in this report.

## Conclusion

The responses to the COVID-19 pandemic from individual state chiropractic licensing boards were heterogenous and, in many cases, provided little or no guidance regarding changes to chiropractic practice during the COVID-19 pandemic. State chiropractic licensing boards have an implicit mandate to regulate chiropractic practice and protect the public within their respective states. A minority of states collated important COVID-19-related guidance and information in a single locale, either on or linked directly from their state board’s website. The authors of this report consider assembling information into a single publicly available location, displaying the time and date of last update, and highlighting its availability in a central location to be a best practice for communication during emergency situations. We recommend each state board consider adopting this approach to improve delivery of critical information so that relevant changes to practice can be implemented efficiently and universally.

## Supplementary information

**Additional file 1.**

## Data Availability

All data generated or analysed during this study are included in this published article [and its supplementary information files.
