# Supplementary material for: Guidance concerning chiropractic practice in response to COVID-19 in the U.S.: a summary of state regulators’ web-based information
Source: Chiropr Man Therap. 2020 Jul 6;28:44. doi: 10.1186/s12998-020-00333-6 (PMC7336092; doi:10.1186/s12998-020-00333-6)
Supplement: Supplementary file 1 — Additional file 1. [file 12998_2020_333_MOESM1_ESM.docx]

**Appendix A**

State by state reference listing

Alabama:

1. April 2020. Office of the Governor of Alabama. <https://governor.alabama.gov/newsroom/2020/04/>. Accessed 10 Apr 2020.

2. Alabama State Board of Chiropractic Examiners. <http://www.chiro.state.al.us/>. Accessed 10 Apr 2020.

Alaska:

1. Amended Covid-19 Mandate. <https://gov.alaska.gov/wp-content/uploads/sites/2/04072020-COVID-Mandate-005-Revised-Elective-Non-Urgent-Procedures.pdf>. Accessed 10 Apr 2020.

2. Mike Dunleavy, Governor of Alaska Homepage. <https://gov.alaska.gov/>. Accessed 10 Apr 2020.

3. Alaska Board of Chiropractic Examiners . <https://www.commerce.alaska.gov/web/cbpl/ProfessionalLicensing/BoardofChiropracticExaminers/ApprovedContinuingEducationCourses.aspx>. Accessed 10 Apr 2020.

Arizona:

1. Office of the Arizona Governor Doug Ducey. Office of the Arizona Governor Doug Ducey. <https://azgovernor.gov/home>. Accessed 10 Apr 2020.

2. Board of Chiropractic Examiners | State of Arizona. <https://chiroboard.az.gov/>. Accessed 10 Apr 2020.

Arkansas:

1. ChiropracticCOVID19Guidance3.23.20. Arkansas Department of Health . <http://www.arkansas.gov/asbce/applicants_licensees/ChiropracticCOVID19Guidance3.23.20.pdf>. Accessed 10 Apr 2020.

2. Home. <http://www.arkansas.gov/asbce/>. Accessed 10 Apr 2020.

3. Rules and Regulations Arkansas Department of Health. <https://www.healthy.arkansas.gov/rules-and-regulations>. Accessed 10 Apr 2020.

4. Arkansas Governor Asa Hutchinson. <https://governor.arkansas.gov/>. Accessed 10 Apr 2020.

California:

1. Executive Order N-33-20. <https://www.gov.ca.gov/wp-content/uploads/2020/03/3.19.20-attested-EO-N-33-20-COVID-19-HEALTH-ORDER.pdf>. Accessed 10 Apr 2020.

2. California Governor. California Governor. <https://www.gov.ca.gov/>. Accessed 10 Apr 2020.

3. Board of Chiropractic Examiners, California Department of Consumer Affairs. <https://www.chiro.ca.gov/>. Accessed 10 Apr 2020.

4. Board of Chiropractic Examiners Impacts of Executive Order N-39-20 - Board of Chiropractic Examiners. <https://www.chiro.ca.gov/about_us/executive_order_impacts.shtml>. Accessed 10 Apr 2020.

5. Statement on COVID-19 (Coronavirus) - Board of Chiropractic Examiners. <https://www.chiro.ca.gov/about_us/covid19_statement.shtml>. Accessed 10 Apr 2020.

Delaware:

1. Untitled. COVID 19. <https://www.dechiro.com/wp-content/uploads/2020/04/COVID-19.pdf>. Accessed 10 Apr 2020.

2. Board of Chiropractic. Division of Professional Regulation - State of Delaware. <https://dpr.delaware.gov/boards/chiropractic/>. Accessed 10 Apr 2020.

3. Coronavirus. Delaware’s Coronavirus Official Website. <https://coronavirus.delaware.gov/>. Accessed 10 Apr 2020.

4. Governor John Carney - State of Delaware. Governor John Carney - State of Delaware. <https://governor.delaware.gov/>. Accessed 10 Apr 2020.

Connecticut:

1. Coronavirus. CT.gov - Connecticut’s Official State Website. <http://portal.ct.gov/Coronavirus>. Accessed 10 Apr 2020.

2. Emergency Orders issued by the Governor and State Agencies. CT.gov - Connecticut’s Official State Website. <http://portal.ct.gov/Coronavirus/Pages/Emergency-Orders-issued-by-the-Governor-and-State-Agencies>. Accessed 10 Apr 2020.

3. Connecticut Governor Ned Lamont. CT.gov - Connecticut’s Official State Website. <http://portal.ct.gov/governor>. Accessed 10 Apr 2020.

4. Safe Workplace Rules for Essential Employers. CT.gov - Connecticut’s Official State Website. <http://portal.ct.gov/DECD/Content/Coronavirus-Business-Recovery/Safe-Workplace-Rules-for-Essential-Employers>. Accessed 10 Apr 2020.

5. State Board of Chiropractic Examiners. CT.gov - Connecticut’s Official State Website. <http://portal.ct.gov/DPH/Public-Health-Hearing-Office/State-Board-of-Chiropractic-Examiners/State-Board-of-Chiropractic-Examiners>. Accessed 10 Apr 2020.

Georgia:

1. 2020 Executive Orders. Governor Brian P. Kemp Office of the Governor. <https://gov.georgia.gov/executive-action/executive-orders/2020-executive-orders>. Accessed 10 Apr 2020.

2. Plb | Licensing. <https://sos.ga.gov/index.php/licensing/plb/14>. Accessed 10 Apr 2020.

3. CDC. Coronavirus Disease 2019 (COVID-19). Centers for Disease Control and Prevention. 2020. <https://www.cdc.gov/coronavirus/2019-ncov/hcp/ambulatory-care-settings.html>. Accessed 10 Apr 2020.

4. CDC. Coronavirus Disease 2019 (COVID-19). Centers for Disease Control and Prevention. 2020. <https://www.cdc.gov/coronavirus/2019-ncov/hcp/index.html>. Accessed 10 Apr 2020.

Colorado:

1. COVID-19 Updates for Licensees and Consumers. Department of Regulatory Agencies. 2020. <https://www.colorado.gov/pacific/dora/covid-19-updates-licensees-and-consumers>. Accessed 10 Apr 2020.

2. Updated: Guidance for Chiropractic Procedures. Colorado Department of Regulatory Agencies. <https://content.govdelivery.com/accounts/CODORA/bulletins/28527b0>. Accessed 10 Apr 2020.

3. Guidance for Chiropractic Procedures. Colorado Department of Regulatory Agencies. <https://content.govdelivery.com/accounts/CODORA/bulletins/28499a6>. Accessed 10 Apr 2020.

4. Prohibition on Chiropractic Elective Procedures. Colorado Department of Regulatory Agencies. <https://content.govdelivery.com/accounts/CODORA/bulletins/28369d2>. Accessed 10 Apr 2020.

5. Home | Colorado.gov. <https://www.colorado.gov/>. Accessed 10 Apr 2020.

6. Colorado Board of Chiropractic Examiners - Colorado Chiropractic Association. <https://www.coloradochiropractic.org/page/CBCE>. Accessed 10 Apr 2020.

Florida:

1. Medicine FB of C. Florida Board of Chiropractic Medicine- Licensing, Renewals & Information. <https://floridaschiropracticmedicine.gov/>. Accessed 10 Apr 2020.

2. Florida Governor Ron DeSantis. <https://www.flgov.com/>. Accessed 10 Apr 2020.

Hawaii:

1. David Y. Ige. [https://governor.hawaii.gov](https://governor.hawaii.gov/). Accessed 10 Apr 2020.

2. COVID-19 Joint Information Center Updates. <https://health.hawaii.gov/news/covid-19-updates/>. Accessed 10 Apr 2020.

3. Hawaii Board of Chiropractic. <https://cca.hawaii.gov/pvl/boards/chiropractor/>. Accessed 10 Apr 2020.

4. Hawaii Board of Chiropractic. <https://cca.hawaii.gov/pvl/boards/chiropractor/hawaii-board-of-chiropractic/>. Accessed 10 Apr 2020.

Idaho:

1. Idaho A. Governor Brad Little. Office of the Governor. <https://gov.idaho.gov/>. Accessed 10 Apr 2020.

2. Idaho A. Statewide Stay-Home Order. Novel Coronavirus (COVID-19). <https://coronavirus.idaho.gov/statewide-stay-home-order/>. Accessed 10 Apr 2020.

3. Idaho (USA) S of. ibol.Idaho.gov. <https://ibol.idaho.gov/IBOL/BoardPage.aspx?Bureau=CHI>. Accessed 10 Apr 2020.

Illinois:

1. Executive Order 2020-10 - Illinois.gov. [https://www2.illinois.gov:443/Pages/Executive-Orders/ExecutiveOrder2020-10.aspx](https://www2.illinois.gov/Pages/Executive-Orders/ExecutiveOrder2020-10.aspx). Accessed 10 Apr 2020.

2. COVID-19 Executive Order NO.8. <https://www2.illinois.gov/Documents/ExecOrders/2020/ExecutiveOrder-2020-10.pdf>. Accessed 10 Apr 2020.

3. Telehealth Resources | Health Information Technology | ACP. <https://www.acponline.org/practice-resources/business-resources/health-information-technology/telehealth?utm_campaign=FY19-20_MD_TELEHEALTH_EML_CURRICULUM_MD9191_3A&utm_medium=email&utm_source=Eloqua&elqTrackId=59052d1d27704688a4d31f303180da66&elq=4857b1325cfe4ce5bd816bf95e3ed345&elqaid=5180&elqat=1&elqCampaignId=2289>. Accessed 10 Apr 2020.

4. State of Illinois | Department of Financial & Professional Regulation. <https://www.idfpr.com/>. Accessed 10 Apr 2020.

5. Illinois Department of Financial and Professional Regulation Announces Measures to Assist Licensees and Education Providers During the COVID-19 Pandemic. <https://www.idfpr.com/News/2020/2020%2003%2018%20IDFPR%20Variance%20Press%20Release.pdf>. Accessed 10 Apr 2020.

6. JB Pritzker, Governor. <https://www2.illinois.gov/sites/gov/Pages/default.aspx>. Accessed 10 Apr 2020.

Indiana:

1. Executive order 20-08. <https://www.in.gov/gov/files/Executive_Order_20-08_Stay_at_Home.pdf>. Accessed 10 Apr 2020.

2. Governor Eric J. Holcomb: Governor Holcomb. <https://www.in.gov/gov/>. Accessed 10 Apr 2020.

3. ISDH - Novel Coronavirus: Indiana Essential Businesses and Operations List. <https://www.coronavirus.in.gov/2496.htm>. Accessed 10 Apr 2020.

4. ISDH - Novel Coronavirus: Professional Resources. <https://www.coronavirus.in.gov/2399.htm>. Accessed 10 Apr 2020.

5. CDC. Coronavirus Disease 2019 (COVID-19). Centers for Disease Control and Prevention. 2020. <https://www.cdc.gov/coronavirus/2019-ncov/hcp/ppe-strategy/index.html>. Accessed 10 Apr 2020.

6. PLA: COVID-19 Actions. <https://www.in.gov/pla/4092.htm>. Accessed 10 Apr 2020.

7. PLA: Home. <https://www.in.gov/pla/>. Accessed 10 Apr 2020.

Iowa:

1. Iowa Board of Chiropractic . <https://idph.iowa.gov/Licensure/Iowa-Board-of-Chiropractic>. Accessed 10 Apr 2020.

2. Proclamation of Disaster Emergency . <https://governor.iowa.gov/sites/default/files/documents/Public%20Health%20Proclamation%20-%202020.04.06.pdf>. Accessed 10 Apr 2020.

3.Office of the Governor of Iowa. <https://governor.iowa.gov/>. Accessed 10 Apr 2020.

Kansas:

1. Execcutive order NO.20-16. <https://governor.kansas.gov/wp-content/uploads/2020/03/EO20-16.pdf>. Accessed 10 Apr 2020.

2. Home. Governor of the State of Kansas. <https://governor.kansas.gov/>. Accessed 1Apr 2020.

3. <https://www.coronavirus.kdheks.gov/DocumentCenter/View/364/Outpatient-Clinic-COVID-19-Readiness-PDF---3-26-20?bidId=>. <https://www.coronavirus.kdheks.gov/DocumentCenter/View/364/Outpatient-Clinic-COVID-19-Readiness-PDF---3-26-20?bidId=>. Accessed 10 Apr 2020.

4. CDC. Coronavirus Disease 2019 (COVID-19). Centers for Disease Control and Prevention. 2020. <https://www.cdc.gov/coronavirus/2019-ncov/hcp/index.html>. Accessed 10 Apr 2020.

5. Healthcare Providers & Local Health Departments | KDHE COVID-19. <https://www.coronavirus.kdheks.gov/170/Healthcare-Providers>. Accessed 10 Apr 2020.

6. Kansas State Board of Healing Arts. <http://www.ksbha.org/main.shtml>. Accessed 10 Apr 2020.

Kentucky:

1. Executive order 2020-257. <https://governor.ky.gov/attachments/20200325_Executive-Order_2020-257_Healthy-at-Home.pdf>. Accessed 10 Apr 2020.

2. Welcome - Kentucky Governor Andy Beshear. <https://governor.ky.gov/>. Accessed 10 Apr 2020.

3. Executive order 2020-246. <https://governor.ky.gov/attachments/20200322_Executive-Order_2020-246_Retail.pdf>. Accessed 10 Apr 2020.

4. Kentucky Board of Chiropractic Examiners Welcome. <https://kbce.ky.gov/Pages/default.aspx>. Accessed 10 Apr 2020.

Louisiana:

1. Proclomation number 33 JBE 2020. <https://gov.louisiana.gov/assets/Proclamations/2020/JBE-33-2020.pdf>. Accessed 10 Apr 2020.

2. Office of Governor John Bel Edwards. <https://gov.louisiana.gov/>. Accessed 10 Apr 2020.

3. COVID-19 Statewide Stay at Home Order. <https://gov.louisiana.gov/assets/docs/covid/Essential-Infrastructure_fact-sheet.pdf>. Accessed 10 Apr 2020.

4. What’s New. <http://www.lachiropracticboard.com/whatsnew.htm>. Accessed 10 Apr 2020.

5. What’s New. <http://www.lachiropracticboard.com/whatsnew.htm>. Accessed 10 Apr 2020.

Maine:

1. Home | Office of Governor Janet T. Mills. <http://www.maine.gov/governor/mills/>. Accessed 10 Apr 2020.

2. Maine PFR - Professions - Chiropractic Licensure. <https://www.maine.gov/pfr/professionallicensing/professions/chiropractors/index.html>. Accessed 10 Apr 2020.

Maryland:

1. Home. The Office of Governor Larry Hogan. <http://governor.maryland.gov/>. Accessed 10 Apr 2020.

2. State Board of Chiropractic Examiners. [https://health.maryland.gov/chiropractic/Pages/index.aspx. Accessed 10 Apr 2020](https://health.maryland.gov/chiropractic/Pages/index.aspx.%20Accessed%2010%20Apr%202020).

Minnesota:

1. Office of Governor. Office of Governor Tim Walz and Lt. Governor Peggy Flanagan. <https://mn.gov/governor/>. Accessed 10 Apr 2020.

2. Chiropractic Examiners, Minnesota Board of. Minnesota Board of Chiropractic Examiners. <https://mn.gov/boards/chiropractic-examiners/>. Accessed 10 Apr 2020.

Mississippi:

1. Mississippi Board of Chiropractic Examiners - > Home. <https://www.msbce.ms.gov/secure/index.asp>. Accessed 29 Apr 2020.

2. Welcome. Office of Governor Tate Reeves. <https://governorreeves.ms.gov/>. Accessed 10 Apr 2020.

Missouri:

1. Stay at Home Order | Governor Michael L. Parson. <https://governor.mo.gov/priorities/stay-home-order>. Accessed 10 Apr 2020.

2. Home page | Governor Michael L. Parson. <https://governor.mo.gov/>. Accessed 10 Apr 2020.

3. “Stay Home Missouri” Order - Guidance and Frequently Asked Questions | Governor Michael L. Parson. <https://governor.mo.gov/stay-home-missouri-order-guidance-and-frequently-asked-questions>. Accessed 10 Apr 2020.

4. Missouri Board of Chiropractic Examiners . <https://pr.mo.gov/chiropractors.asp>. Accessed 10 Apr 2020.

Montana:

1. Montana Chiropractic Association | Working to Advance Chiropractic Care. Montana Chiropractic Association. <http://mtchiro.org/>. Accessed 10 Apr 2020.

2. Board of Chiropractors. <http://boards.bsd.dli.mt.gov/chi#0>(. Accessed 10 Apr 2020.

3. STATE OF MONTANA OFFICE OF THE GOVERNOR EXECUTIVE ORDER No. 2-2020. <https://covid19.mt.gov/Portals/223/Documents/EO-02-2020_COVID-19%20Emergency%20Declaration.pdf?ver=2020-03-13-072730-880>. Accessed 10 Apr 2020.

4. Directive Implementing Executive Orders 2-2020 and 3-2020 providing measures to stay at home and designating certain essential functions. <https://covid19.mt.gov/Portals/223/Documents/Stay%20at%20Home%20Directive.pdf?ver=2020-03-26-173332-177>. Accessed 10 Apr 2020.

5. Governor Steve Bullock - State of Montana > Home. <https://governor.mt.gov/>. Accessed 10 Apr 2020.

Nebraska:

1. Latest News | Office of Governor Pete Ricketts. <https://governor.nebraska.gov/>. Accessed 10 Apr 2020.

2. Chiropractic. [http://dhhs.ne.gov:80/licensure/Pages/Chiropractic.aspx](http://dhhs.ne.gov/licensure/Pages/Chiropractic.aspx). Accessed 10 Apr 2020.

Nevada:

1. Chiropractic Physicians’ Board of Nevada. <http://chirobd.nv.gov/>. Accessed 10 Apr 2020.

2. Untitled. <http://dpbh.nv.gov/>. Accessed 10 Apr 2020.

3. Nevada Governor. <http://gov.nv.gov/>. Accessed 10 Apr 2020.

Massachusetts:

1. Board of Registration of Chiropractors. Mass.gov. <https://www.mass.gov/orgs/board-of-registration-of-chiropractors>. Accessed 10 Apr 2020.

2. Office of Governor Charlie Baker and Lt. Governor Karyn Polito. Mass.gov. <https://www.mass.gov/orgs/office-of-the-governor>. Accessed 10 Apr 2020.

New Hampshire:

1. Emergency Order # 17 Pursuant to Executive Order 2020-04. <https://www.governor.nh.gov/news-media/emergency-orders/documents/emergency-order-17-1.pdf>. Accessed 10 Apr 2020.

2. Welcome to the Office of the Governor | Governor Christopher T. Sununu. <https://www.governor.nh.gov/>. Accessed 10 Apr 2020.

3. Board of Chiropractic Examiners | Office of Professional Licensure and Certification. <https://www.oplc.nh.gov/chiropractic/>. Accessed 10 Apr 2020.

New Jersey:

1. State Board of Chiropractic Examiners. <https://www.njconsumeraffairs.gov/chi/Pages/default.aspx>. Accessed 10 Apr 2020.

2. New Jersey COVID-19 Information Hub. <https://covid19.nj.gov/>. Accessed 10 Apr 2020.

3. The Official Web Site for The State of New Jersey. <https://nj.gov/>. Accessed 10 Apr 2020.

New Mexico

1. State enacts further restrictions to stop spread, including stay-at-home instruction | Welcome to NewMexico.gov. <https://www.newmexico.gov/2020/03/23/state-enacts-further-restrictions-to-stop-spread-including-stay-at-home-instruction/>. Accessed 10 Apr 2020.

2. Chiropractic Board. <http://www.rld.state.nm.us/boards/chiropractic-board.aspx>. Accessed 10 Apr 2020.

New York:

1. New York State on PAUSE. Department of Health. <https://coronavirus.health.ny.gov/new-york-state-pause>. Accessed 10 Apr 2020.

2. COVID-19 FAQs. [http://www.op.nysed.gov/COVID-19FAQS.html. Accessed 10 Apr 2020](http://www.op.nysed.gov/COVID-19FAQS.html.%20Accessed%2010%20Apr%202020).

3. COVID-19. <http://www.op.nysed.gov/COVID-19.html>. Accessed 10 Apr 2020.

4. NYS Chiropractic. <http://www.op.nysed.gov/prof/chiro/>. Accessed 10 Apr 2020.

North Carolina:

1. NC Governor Roy Cooper. <https://governor.nc.gov/>. Accessed 10 Apr 2020.

2. North Carolina Board of Chiropractic Examiners. NCBOCE. <https://ncchiroboard.com/>. Accessed 10 Apr 2020.

North Dakota:

1. Home. North Dakota Office of the Governor. <https://www.governor.nd.gov/home>. Accessed 10 Apr 2020.

2. State of ND COVID-19 (Coronavirus) Resources. ND Response. <https://ndresponse.gov/covid-19-resources>. Accessed 10 Apr 2020.

3. Executive Orders. North Dakota Office of the Governor. <https://www.governor.nd.gov/executive-orders>. Accessed 10 Apr 2020.

4. Home - ND Board of Chiropractic Examiners. <https://www.ndsbce.org/>. Accessed 10 Apr 2020.

Ohio:

1. Ohio Issues “Stay at Home” Order; New Restrictions Placed on Day Cares for Children. <https://governor.ohio.gov/wps/portal/gov/governor/media/news-and-media/ohio-issues-stay-at-home-order-and-new-restrictions-placed-on-day-cares-for-children?mod=article_inline>. Accessed 10 Apr 2020.

2. Board guidance . Ohio state chiropractic board . [https://chirobd.ohio.gov/COVID-19/3192020BoardGuidance.aspx. Accessed 10 Apr 2020](https://chirobd.ohio.gov/COVID-19/3192020BoardGuidance.aspx.%20Accessed%2010%20Apr%202020).

Oklahoma:

1. Oklahoma Board of Chiropractic Examiners - Home. <https://www.ok.gov/chiropracticboard/>. Accessed 10 Apr 2020.

2. Office of Governor | Kevin Stitt. <https://www.governor.ok.gov/>. Accessed 10 Apr 2020.

Oregon:

1. Oregon NewsRoom . Governor’s Office . <https://www.oregon.gov/newsroom/Pages/Agency.aspx?agency=GOV>. Accessed 10 Apr 2020.

2. State of Oregon : Oregon.gov Home Page : State of Oregon. <https://www.oregon.gov/Pages/index.aspx>. Accessed 10 Apr 2020.

3. Oregon Board of Chiropractic Examiners : COVID-19 Announcements : State of Oregon. <https://www.oregon.gov/obce/Pages/COVID-19_Announcements.aspx>. Accessed 10 Apr 2020.

4. Oregon Board of Chiropractic Examiners : Welcome Page : State of Oregon. <https://www.oregon.gov/OBCE/Pages/index.aspx>. Accessed 10 Apr 2020.

Pennsylvania:

1. Home. Pennsylvania Department of State. [https://www.dos.pa.gov:443/ProfessionalLicensing/BoardsCommissions/Chiropractic/Pages/default.aspx](https://www.dos.pa.gov/ProfessionalLicensing/BoardsCommissions/Chiropractic/Pages/default.aspx). Accessed 10 Apr 2020.

2. Pennsylvania Governor Tom Wolf. Governor Tom Wolf. <https://www.governor.pa.gov/>. Accessed 10 Apr 2020.

Rhode Island:

1. Executive Orders . Office of the Governor . <https://governor.ri.gov/newsroom/orders/>. Accessed 10 Apr 2020.

2. Untitled. Office of the Governor . <https://governor.ri.gov/>. Accessed 10 Apr 2020.

3. Chiropractic Licensing: Department of Health. <https://health.ri.gov/licenses/detail.php?id=250>. Accessed 10 Apr 2020.

South Dakota:

1. Coronavirus updates and information. <https://doh.sd.gov/news/Coronavirus.aspx>. Accessed 10 Apr 2020.

2. State of South Dakota Governor’s Website. <https://sd.gov/governor/>. Accessed 10 Apr 2020.

3. South Dakota Board of Chiropractic Examiners - SD Dept. of Health. <https://doh.sd.gov/boards/chiropractic/>. Accessed 10 Apr 2020.

Tennessee:

1. Letter to health care providers . <https://www.tn.gov/content/dam/tn/health/healthprofboards/Commissioner-Piercey-COVID-19-letter.pdf>. Accessed 10 Apr 2020.

2. Executive order NO. 23. <https://publications.tnsosfiles.com/pub/execorders/exec-orders-lee23.pdf>. Accessed 10 Apr 2020.

3. Executive order NO. 21 . <https://publications.tnsosfiles.com/pub/execorders/exec-orders-lee21.pdf>. Accessed 10 Apr 2020.

4. Governor. <https://www.tn.gov/governor.html>. Accessed 10 Apr 2020.

5. Chiropractic Examiners. <https://www.tn.gov/health/health-program-areas/health-professional-boards/chiro-board.html>. Accessed 10 Apr 2020.

Texas:

1. Home. [https://gov.texas.gov](https://gov.texas.gov/). Accessed 10 Apr 2020.

2. Governor Abbott Issues Executive Order, Implements Statewide Essential Services And Activities Protocols. <https://gov.texas.gov/news/post/governor-abbott-issues-executive-order-implements-statewide-essential-services-and-activities-protocols>. Accessed 10 Apr 2020.

3. Texas Board of Chiropractic Examiners. <https://www.tbce.state.tx.us/>. Accessed 10 Apr 2020.

Utah:

1. Governor Gary Herbert | Governor Gary Herbert. <https://governor.utah.gov/>. Accessed 10 Apr 2020.

2. Recommendations for Providers | coronavirus. <https://coronavirus.utah.gov/recommendations-for-providers/>. Accessed 10 Apr 2020.

3. COVID 19. DOPL COVID19. <https://dopl.utah.gov/covid19.html>. Accessed 10 Apr 2020.

Vermont:

1. What Stay Home/Stay Safe Order Means to Vermonters | Vermont Emergency Management. <https://vem.vermont.gov/StayHome>. Accessed 10 Apr 2020.

2. COVID-19: Important OPR Policies & Service Impacts. <https://sos.vermont.gov/opr/about-opr/covid-19-response/#q6>. Accessed 10 Apr 2020.

3. BOARD OF CHIROPRACTIC | Office of Governor Phil Scott. <https://governor.vermont.gov/boards_and_commissions/chiropractic>. Accessed 10 Apr 2020.

Virginia:

1. [webmaster@governor.virginia.gov](mailto:webmaster@governor.virginia.gov). Virginia Governor Ralph Northam - Governor of Virginia. <https://www.governor.virginia.gov/>. Accessed 10 Apr 2020.

2. Virginia Board of Medicine. <https://www.dhp.virginia.gov/medicine/default.htm>. Accessed 10 Apr 2020.

3. Virginia Board of Medicine. <https://www.dhp.virginia.gov/medicine/>. Accessed 10 Apr 2020.

Washington:

1. Governor Jay Inslee | Washington State. <https://www.governor.wa.gov/>. Accessed 10 Apr 2020.

2. Untitled. <https://www.doh.wa.gov/>. Accessed 10 Apr 2020.

3. Chiropractor, Chiropractic X-Ray Technician home page :: Washington State Department of Health. <https://www.doh.wa.gov/LicensesPermitsandCertificates/ProfessionsNewReneworUpdate/Chiropractor>. Accessed 10 Apr 2020.

West Virginia:

1. Updates and News. <https://dhhr.wv.gov/COVID-19/pages/updates-and-news.aspx>. Accessed 10 Apr 2020.

2. Untitled. <https://boc.wv.gov/Documents/Covid-19%20Email%20to%20Chiropractic%20Licensees.pdf>. Accessed 10 Apr 2020.

3. Welcome to the Board of Chiropractic. <https://boc.wv.gov/Pages/default.aspx>. Accessed 10 Apr 2020.

4. Office of the Governor. <https://governor.wv.gov/Pages/default.aspx>. Accessed 10 Apr 2020.

5. Coronavirus Disease 2019 (COVID-19). <https://dhhr.wv.gov/COVID-19/Pages/default.aspx>. Accessed 10 Apr 2020.

Wisconsin:

1. Official site of Governor Tony Evers. <https://evers.wi.gov/Pages/Home.aspx>. Accessed 10 Apr 2020.

2. Mieske J. Essential Business Declaration. WEDC. <https://wedc.org/essentialbusiness/>. Accessed 10 Apr 2020.

3. DSPS Home. <https://dsps.wi.gov/pages/Home.aspx>. Accessed 10 Apr 2020.

4. DSPS Chiropractic Examining Board. <https://dsps.wi.gov/Pages/BoardsCouncils/Chiropractic/Default.aspx>. Accessed 10 Apr 2020.

Wyoming:

1. Home. <https://sites.google.com/wyo.gov/chiropractic>. Accessed 10 Apr 2020.

2. HOME. <https://sites.google.com/wyo.gov/govgordon>. Accessed 10 Apr 2020.
